# Alternatives to Aerobic Exercise Prescription in Patients with Chronic Heart Failure

**DOI:** 10.5935/abc.20160014

**Published:** 2016-02

**Authors:** Mayron F Oliveira, Gabriela Zanussi, Bianca Sprovieri, Denise M. L. Lobo, Luiz E Mastrocolla, Iracema I. K. Umeda, Priscila A Sperandio

**Affiliations:** 1 Setor de Reabilitação Cardiovascular - Equipe de Fisioterapia - Instituto Dante Pazzanese de Cardiologia São Paulo, SP - Brazil; 2 Setor de Reabilitação Cardiovascular - Equipe Médica - Instituto Dante Pazzanese de Cardiologia São Paulo, SP - Brazil

**Keywords:** Exercise Prescription, Chronic Heart Failure, Cardiopulmonary Exercise Test, Six-minute Walk Test, Rehabilitation.

## Abstract

**Background:**

Exercise is essential for patients with heart failure as it leads to a reduction in morbidity and mortality as well as improved functional capacity and oxygen uptake (⩒O_2_). However, the need for an experienced physiologist and the cost of the exam may render the cardiopulmonary exercise test (CPET) unfeasible. Thus, the six-minute walk test (6MWT) and step test (ST) may be alternatives for exercise prescription.

**Objective:**

The aim was to correlate heart rate (HR) during the 6MWT and ST with HR at the anaerobic threshold (HR_AT_) and peak HR (HR_P_) obtained on the CPET.

**Methods:**

Eighty-three patients (58 ± 11 years) with heart failure (NYHA class II) were included and all subjects had optimized medication for at least 3 months. Evaluations involved CPET (⩒O_2_, HR_AT_, HR_P_), 6MWT (HR_6MWT_) and ST (HR_ST_).

**Results:**

The participants exhibited severe ventricular dysfunction (ejection fraction: 31 ± 7%) and low peak ⩒O2 (15.2 ± 3.1 mL.kg_-1_.min_-1_). HR_P_ (113 ± 19 bpm) was higher than HR_AT_ (92 ± 14 bpm; p < 0.05) and HR_6MWT_ (94 ± 13 bpm; p < 0.05). No significant difference was found between HR_P_ and HR_ST_. Moreover, a strong correlation was found between HR_AT_ and HR_6MWT_ (r = 0.81; p < 0.0001), and between HR_P_ and HR_ST_ (r = 0.89; p < 0.0001).

**Conclusion:**

These findings suggest that, in the absence of CPET, exercise prescription can be performed by use of 6MWT and ST, based on HR_6MWT_ and HR_ST_

## Introduction

Heart failure (HF) is a complex systemic condition. In recent years, consistent scientific evidence has indicated that aerobic physical exercise is an effective non-pharmacological treatment strategy.^1-3^ Determination of exercise intensity is the most important factor in achieving benefits while maintaining a safe level of cardiovascular rehabilitation.^4,5^ To that end, the cardiopulmonary exercise test (CPET) is the gold standard for maximum aerobic exercise intensity prescription.^6,7^ This test provides objective measures of metabolic, respiratory, and cardiovascular responses at anaerobic threshold and respiratory compensation point.^6^ However, the CPET is not always available at cardiovascular rehabilitation centers.

A number of formulas for predicting maximum and training heart rate (HR) have been proposed in the literature,^8,9^ as it is an easy and inexpensive way of monitoring and prescribing aerobic exercise.^4^ However, those formulas have been developed in an arbitrary fashion and their effectiveness has not been proven using scientific criteria.^10^ Moreover, none of the formulas are specific to the HF population or take into consideration medications used by these patients. Thus, alternative exercise prescription methods are needed for HF patients.

In the absence of the CPET, the six-minute walk test (6MWT) and step test (ST) constitute alternatives for evaluating HF patients. The 6MWT is a simple, low-cost, easily administered method of evaluating submaximal capacity.^11-13^ The ST requires minimal physical space, and evidence presented in recent years has revealed its usefulness in estimating exercise tolerance.^14^ The ST is classified as a maximum or nearly maximum capacity test for moderate to severe HF.^15,16^

While 6MWT and ST are used consistently for the evaluation of functional capacity and exercise tolerance in patients with HF, the reliability of exercise prescriptions based on these tests has been widely questioned. Considering the 6MWT as a submaximal test and the ST as a maximum test in this population, we hypothesized that HR at the anaerobic threshold can be determined by the 6MWT and that peak HR can be determined by the ST, thus allowing for a trustworthy exercise prescription for HF patients when it is not possible to perform the CPET.

## Methods

A cross-sectional study was carried out involving 83 sedentary patients recruited from the Cardiovascular Rehabilitation Unit of Dante Pazzanese Institute of Cardiology, São Paulo, Brazil. All of the patients had left ventricle ejection fraction < 40% and were classified as functional class II [New York Heart Association (NYHA)]. They were stable, with optimal treatment that included beta-blockers (carvedilol, maximum dosage 50 mg/day), angiotensin-converting enzyme inhibitors or angiotensin-receptor blockers and diuretics. None of the patients had undergone cardiac resynchronization therapy or had a left ventricular assistance device. Patients with clinical and/or functional evidence of chronic expiratory flow limitation (FEV_1_/FVC < 0.7; FEV_1_: forced expiratory volume in the first second; FVC: forced vital capacity), smoking habit, unstable angina, significant cardiac arrhythmia, pacemaker, atrial fibrillation, myocardial infarction within the previous 12 months, or participation in cardiac rehabilitation (within 6 months) were excluded. All participants provided written informed consent, and the study protocol was approved by the institutional ethics research committee (nº 4093).

### Study Protocol

All patients performed an individualized ramp-incremental exercise test to determine the difference between HR at the anaerobic threshold (HR_AT_) and HR at peak exercise (HR_P_). On different days, they performed the 6MWT and the ST to determine HR at the end of the tests (HR_6MWT_ and HR_ST_, respectively). All tests were randomized and performed in the morning, with a minimum 48-hour interval. The medications were maintained.

### Cardiopulmonary exercise testing

CPET were performed on an ATL treadmill (Inbramed, Porto Alegre, Brazil) with breath-by-breath variables measured using a commercially available metabolic cart (ULTIMA System^TM^; MGC - USA). Heart rate was continuously monitored using a 12-lead electrocardiogram, and oxyhemoglobin saturation was determined by pulse oximetry (SpO_2_, %; Nonin^TM^ portable oximeter - USA). The subjects were asked to rate their sensations of shortness of breath and leg discomfort at the end of the CPET using the modified Borg scale of perceived exertion (0 to 10).^17^ Spirometric tests were performed before CPET.

Anaerobic threshold was determined by V-slope method, that means the break point between carbon dioxide and oxygen uptake (⩒O_2_) increase or measured by ventilatory equivalent for oxygen and end-tidal carbon dioxide partial pressure. The maximal exercise capacity, peak ⩒O_2_, was determined as the maximum ⩒O_2_ attained at the end of CPET - when the patient could not perform cycle ergometer velocity at 60 rpm.^18-20^

### Six-minute walk test and step test

The 6MWT was performed following the guidelines of the American Thoracic Society.^21^ Before and after the test, blood pressure (BP) (Unilec^TM^ sphygmomanometer and Littmann Quality stethoscope - USA), HR (Polar^®^ RS800 - Polar Electro OY, Finland) and SpO_2 _(Nonin^TM^ portable oximeter - USA) were measured. Heart rate and SpO_2_ were continuously measured during the test, and the modified Borg scale of perceived exertion was used at the end of the test.

The duration of the ST was 4 minutes. Patients were instructed to go up and down a 0.20 m high single-step platform with no handrails and to perform the test at a velocity within their own limitations. The examiner offered verbal stimulation to encourage and to inform the participant regarding test performance. Heart rate and SpO_2_ were measured continuously during the test. The modified Borg scale of perceived exertion was used and BP was measured before and after the test, as well as 2 minutes after recovery.

### Statistical analysis

Statistical analysis was carried out using the SPSS program (version 15.0; SPSS Inc.) The data are expressed as mean ± standard deviation and percentage. The Kolmogorov-Smirnov test was used to determine the normality of the data distribution. The t-test was used for related samples, and Pearson's ρ, for correlations between variables. Both slope and intercept were examined. In addition, a Bland-Altman plot was used to examine HR variables. Moreover, standard error of estimate (SEE) was applied for HR_6MWT_ and HR_AT_and for HR_ST_ and HR_P_. For all analyses, statistical significance was set at 5% (p < 0.05).

## Results

Eighty-three patients with HF were enrolled in the study (Table 1). None of the patients had spirometric signs of chronic obstructive pulmonary disease (FVC: 84.9 ± 10.3% predicted; FEV_1_: 80.3 ± 13.2% predicted; FEV_1_/FVC: 0.78 ± 0.12) or exhibited any criteria for CPET, 6MWT, or ST interruption (ventricular arrhythmia, arterial pressure drop, low SpO_2_, or signs of lower cardiac output).

**Table 1:** Characteristics of 83 patients with chronic heart failure

Anthropometrics/Demographics	
Male/Female, n	65/18
Age, years	58 ± 11
Weight, kg	76.7 ± 12.5
Height, m	1.64 ± 9.4
BMI, kg/m^2^	26.7 ± 6.2
LVEF, %	31 ± 7
**Main comorbidities**	
Hypertension, n (%)	60 (72.3%)
Dyslipidemia, n (%)	56 (67.5%)
Diabetes mellitus, n (%)	23 (27.7%)
**Etiology**	
Ischemic, n (%)	62 (74.7%)
Non-ischemic, n (%)	14 (16.9%)
Chagasic, n (%)	7 (8.4%)
**Main medications**	
β-blocker, n (%)	83 (100%)
ACE inhibitors or ARBs, n (%)	83 (100%)
Diuretics, n (%)	83 (100%)

kg: kilogram; m: meters; BMI: body mass index; LVEF: left ventricular ejection fraction; ACE: angiotensin-converting enzyme; ARBs: angiotensin II receptor blockers. Values are expressed as mean ± standard deviation or frequency (n).

The patients exhibited low ⩒O_2_ during peak exercise and an extremely reduced O_2_ uptake efficiency slope (Table 2). On the CPET, HR_P_ was higher than HR_AT _(113 ± 19 bpm vs. 92 ± 14 bpm, respectively; p < 0.05) and HR_6MWT_ (94 ± 13; p < 0.05), but no statistically significant difference was found between HR_P_ and HR_ST_ (113 ± 19 bpm vs. 110 ± 17 bpm; p > 0.05). There was also no significant difference between HR_AT_ and HR_6MWT_. The percentages of predicted HR for HR_AT_ and HR_6MWT_ were similar, as well as the percentages of predicted HR for HR_P_ and HR_ST_ (Table 2).

**Table 2:** Cardiopulmonary Exercise Test (CPET), six-minute walk test (6MWT) and step test (ST)

Cardiopulmonary Exercise Test	
⩒O_2_ peak (mL.kg^-1^.min^-1^)	15.2 ± 3.1
⩒O_2_ peak (% predicted)	28.9 ± 5.0
RER	1.12 ± 0.09
⩒E/⩒CO_2_ Slope	37.7 ± 7.9
O_2_ uptake efficiency slope	1204.5 ± 25.9
O_2_ Pulse (mL/bpm)	10.2 ± 2.6
Rest HR (bpm)	68 ± 11
HR_AT_ (bpm)	92 ± 14
HR_AT_ (% predicted)	55 ± 13
HR_P_ (bpm)	113 ± 19
HR_P_ (% predicted)	70 ± 16
Borg dyspnea	7 ± 2
**Six-minute walk test**	
6MWT (m)	456 ± 83
HR_6MWT_ (bpm)	94 ± 13
HR_6MWT_ (% predicted)	58 ± 10
SBP_6MWT_ (mmHg)	121 ± 18
SpO_2 6MWT_ (%)	96 ± 2
Borg dyspnea	3 ± 1
**Step test**	
Steps (number of steps)	92 ± 20
HR_ST_ (bpm)	110 ± 17
HR_ST_ (% predicted)	67 ± 19
SBP_ST_ (mmHg)	120 ± 23
SpO_2 ST_ (%)	96 ± 1
Borg dyspnea	6 ± 2

⩒O_2_: oxygen uptake; mL: milliliter; kg: Kilogram; min: minute; RER: respiratory exchange ratio; ⩒E: minute ventilation; ⩒CO_2_: carbon dioxide output; O_2_: oxygen; bpm: beats per minute; HR: heart rate; AT: anaerobic threshold; P: peak; m: meters; mmHg: millimeters of Hg; SBP: systolic blood pressure; SpO_2_: oxyhemoglobin saturation. Values are expressed as mean ± standard deviation.

**Figure 1:**
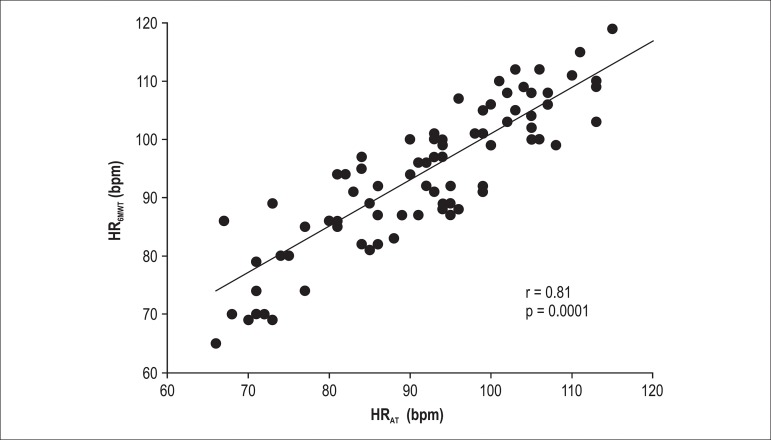
Correlation between HR_AT_ and HR_6MWT_.

**Figure 2:**
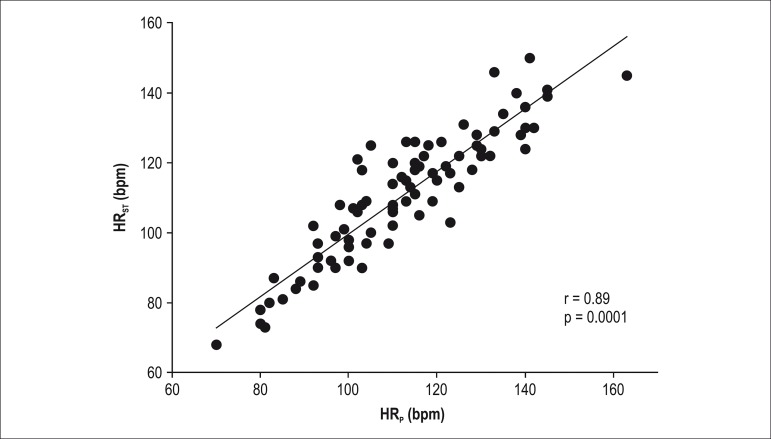
Correlation between HR_P_ and HR_ST_

Significant correlations were found between HR_AT_ and HR_6MWT_ (r = 0.81; p = 0.0001; Figure 1) and between HR_ST_ and HR_P_ (r = 0.89; p = 0.0001; Figure 2) with slope and intercept for HR_AT_ and HR_6MWT _(y = 0.8555x + 15.408; r^2^ = 0.78) and HR_ST_ and HR_P _(y = 0.8947x + 10.28; r^2^ = 0.82). No correlations were found between HR_P_ and HR_6MWT_ (p > 0.05) or between HR_ST_ and HR_AT_ (p > 0.05).

**Figure 3:**
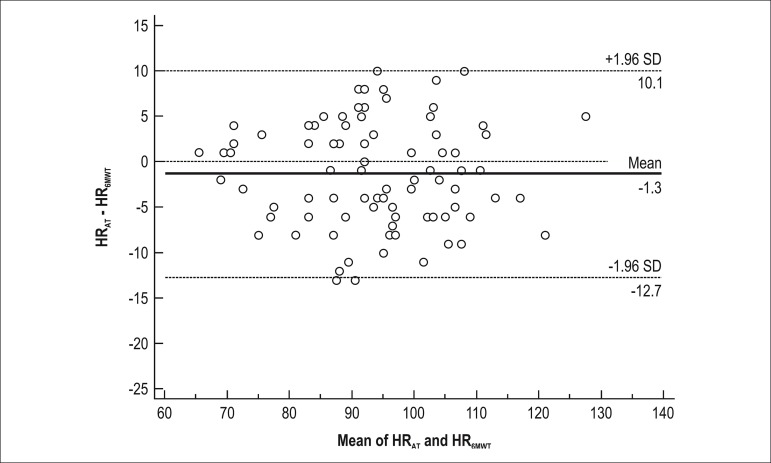
Bland-Altman plot of HR_6MWT_ and HR_AT_.

**Figure 4:**
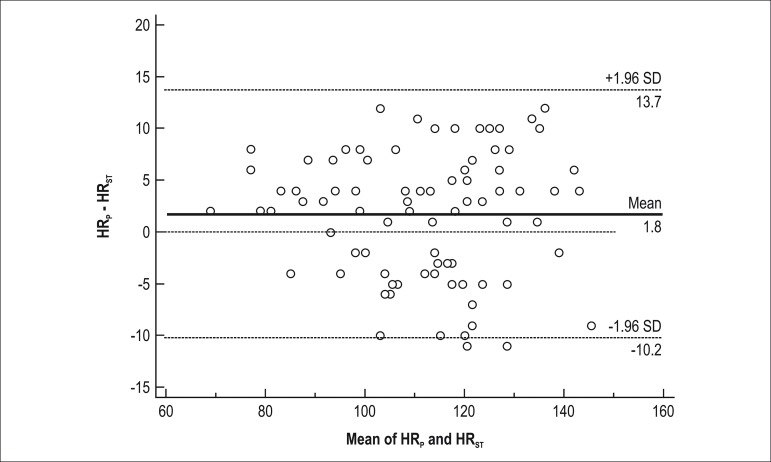
Bland-Altman plot of HR_ST_ and HR_P_.

Despite on variations in HR, Bland-Altman method was used to compare HR_6MWT_ and HR_AT _(Figure 3) and to compare HR_ST_ and HR_P_ (Figure 4). In addition, no differences were found in SEE between HR_AT_ and HR_6MWT_ (SEE = 6.05 bpm) and between HR_P_ and HR_ST _(SEE = 7.69 bpm). Twenty-two patients (26%) showed a difference higher than 5 bpm between HR_AT_ and HR_6MWT_; twenty-three patients (28%) showed a difference higher than 5 bpm between HR_P_ and HR_ST_.

Significant differences in the modified Borg scale of perceived exertion were found between 6MWT and ST, as well as between 6MWT and peak CPET (Table 2). No significant difference was found between ST and CPET. There were no differences in SpO_2_ or BP at the end of the tests between CPET and 6MWT and ST (Table 2).

## Discussion

While CPET is the gold standard for determining HR at anaerobic threshold and at peak exercise, many rehabilitation centers do not have the necessary equipment to perform CPET.^22^ This study demonstrates that aerobic exercise can be prescribed based on 6MWT and ST for patients with clinically stable HF (NYHA class II). Thus, the aim of the present study was to offer alternatives to exercise prescription in patients with HF when CPET cannot be performed. The hallmark findings of this study are that HR_P_ was correlated with HR_ST_, and that HR_AT_ was correlated with HR_6MWT_, enabling exercise prescription based on 6MWT and ST.

Other studies on exercise prescription take into consideration formulas for healthy populations,^9^ and the examiner must choose the most adequate formula for the individual or target population. To facilitate this process, the formula proposed by Fox and Haskell in the 1970s (220 minus age) has been used for a long time to calculate maximum HR.^23,24^ However, it has no validation for chronic HF patients, and it was based only on observations. In the absence of an adequate formula for individuals with disease, a suggestion for exercise prescription was proposed by Cooper (2001)^25^ based on the use of maximal ⩒O_2_ calculated from age, gender, height, and weight. In the present study, however, aerobic exercise prescription was determined without formulas. Moreover, the use of 6MWT and ST for exercise prescription provides a direct measure of the physical condition, HR, BP, and related symptoms (modified Borg scale of perceived exertion) of patients with HF.

In the present study, similar results were found between HR_6MWT_ and HR_AT_, suggesting that the 6MWT is a submaximal test^26,27^ and that a safe exercise prescription can be determined based on the results of this test. The ST literature is scarce, especially with regard to the evaluation of HF patients. A previous study that evaluated exercise capacity in patients and healthy controls based on the results of the CPET and ST found that maximum limits were often achieved on the ST, demonstrating that this is a maximum test for certain populations.^28,29^ The same was found in the present study, in which a strong correlation was demonstrated between HR_ST_ and HR_P_.

According to the American College of Sports Medicine, exercise intensity is considered the most important variable,^30^ and to achieve the benefits provided by the regular practice of physical exercise, exercise prescription should be individual and follow basic principles regarding mode, intensity, frequency, and duration.^4,31^ The American Heart Association recommends at least 30 minutes of moderate aerobic exercise to achieve exercise benefits (at 60%-75% of maximal predicted HR).^32^ On the other hand, exercise can be prescribed between anaerobic threshold and critical power without additional risk.^7,33^ However, determining critical power is extremely complex, and a CPET is mandatory.

Aerobic exercise prescription can be accomplished using HR, and it can be determined using the prescription method proposed in this study, with either moderate-intensity (6MWT) or high-intensity (ST) exercise. Some authors have reported that the 6MWT was related to percentage of ⩒O_2_, corresponding with anaerobic threshold in HF patients,^34,35^ and that HR was closely related to ⩒O_2 _in patients with HF.^36-38^In addition, the American Heart Association and some authors suggest exercising at moderate HR intensities.^32,39,40^ In order to determine exercise intensity based on 6MWT and ST, HR "moderate-load" training should be calculated based on HR_6MWT_, and HR "high-load" training should be based on HR_ST_. The authors suggest two types of exercise prescription using the 6MWT and ST to ideal target of HR: (i) HR_6MWT_ plus 10% (HR_6MWT_ + 10%) or (ii) HR_6MWT_ until HR_ST_ minus 10% (HR_6MWT_ to HR_ST_ - 10%).

Modified Borg scale of perceived exertion exertion is an alternative measure that should be included in exercise prescription. It has been used to control exercise intensity during cardiovascular rehabilitation sessions.^22,41-43^ Some studies have shown that the modified Borg scale of perceived exertion is valid and positively correlated with HR and blood lactate in healthy and chronic HF populations, even on beta-blocker therapy.^41^ However, the criteria for exercise interruption should be followed when the patient reports any symptom or when the value of any variable is above the desired level for the exercise.^4^ Furthermore, HR cannot always be used for prescribing exercises (such as in cases of atrial fibrillation or the inability to perform 6MWT and ST). In such cases, the modified Borg scale of perceived exertion constitutes an alternative for exercise prescription.^7^ In the present study, the modified Borg scale of perceived exertion provided low scores for the 6MWT compared with the ST, demonstrating the usefulness of this scale when HR is not applicable. Moreover, HR monitoring in combination with modified Borg scale of perceived exertion is recommended when prescribing exercises for HF patients on beta-blockers.^38,41^

### Study limitations

The present study has some limitations that should be addressed, due to the small sample size and lack of validation of ST for cardiac patients. This alternative exercise prescription method should be demonstrated in cardiac rehabilitation with different groups of aerobic exercise prescription (CPET prescription vs. 6MWT/ST prescription). However, the present results, albeit obtained in a selected group of stable patients, justify larger longitudinal investigations involving a sizeable number of patients with different NYHA classifications. Other limitations take into account that the tests were not administered in duplicate to ensure reproducibility of the data, and ⩒O_2_ was not measured during the 6MWT or ST. In addition, while significant correlations were found, the method was not administered to the population studied during the rehabilitation process.

## Conclusion

While CPET remains the gold standard for exercise prescription, the present findings suggest a new alternative of exercise prescription for patients with HF based on 6MWT and ST.
